# Enhanced bioavailability of a novel double-layered nano-liposomal curcumin (BNT–C060): a randomized, double-blind, clinical trial

**DOI:** 10.1038/s41598-026-53709-8

**Published:** 2026-05-31

**Authors:** Do-Sung Kim, Hye Kyung Kim, Ki Chan Ha, Gi-Hyun Jang, Se-Ho Park, Do-Hyeon Kim, Yu-Mi Kim, Jaesung Pyo, Jong-Cheon Joo, Han-Jung Chae

**Affiliations:** 1 Non-Clinical Efficacy Evaluation Center, Biomedical Research Institute, Jeonbuk National University Hospital, Jeonju, 54907 Republic of Korea; 2 Research Institute of Clinical Medicine of Jeonbuk National University- Biomedical Research Institute of Jeonbuk National University Hospital, Jeonju, 54907 Republic of Korea; 3 College of Pharmacy and Brain Busan 21 Plus Research Project Group, Kyungsung University, Busan, 48434 Republic of Korea; 4 Healthcare Claims & Management, Inc., Jeonju, 54858 Republic of Korea; 5 Binotec Co., Ltd, 155 Deulan-ro, Suseong-gu, Daegu, Republic of Korea; 6 Department of Sasang Constitutional Medicine, College of Korean Medicine, Wonkwang University, Iksan, 54538 Republic of Korea; 7 School of Pharmacy, Jeonbuk National University, Jeonju, 54907 Republic of Korea

**Keywords:** Curcumin-loaded nano-liposome (BNT–C060), Curcumin bioavailability, Drug delivery system, Pharmacokinetic parameters, Drug discovery, Medical research

## Abstract

Curcumin exhibits potent anti-inflammatory, antioxidant, and neuroprotective effects. However, its therapeutic potential is severely limited by poor oral bioavailability due to low solubility, rapid metabolism, and poor intestinal permeability. This study evaluated the single-dose pharmacokinetics and relative bioavailability of BNT–C060, a novel double-layered chitosan/alginate-coated nano-liposomal curcumin, versus conventional free curcumin in healthy male volunteers. In this randomized, double-blind, parallel-group trial, 18 healthy males (*n* = 9/group) received single oral doses of BNT–C060 (400 mg curcumin-equivalent) or free curcumin (2,000 mg) under fasting conditions. Plasma and urine samples were collected over 24 h for total curcumin analysis (LC–MS/MS after β-glucuronidase treatment). BNT–C060 demonstrated superior dose-normalized pharmacokinetics: 23.2-fold higher plasma AUC_0–t_ (5.00 vs. 0.22 ng·h/mL/mg), 53-fold higher C_max_ (1.06 vs. 0.02 ng/mL/mg), and 35.3-fold higher urinary exposure. T_max_ was earlier (0.86 vs. 3.3 h). A single oral dose of BNT–C060 was generally well tolerated in healthy male volunteers within the evaluated dose range, with no clinically significant safety signals observed under the study conditions. BNT–C060 achieved substantially higher plasma concentrations (C_max_ 423 ng/mL vs. 34 ng/mL free curcumin) compared with free curcumin, suggesting improved systemic exposure with the nano-liposomal formulation. These proof-of-concept findings provide a rationale for further clinical investigation of this advanced nano-liposomal delivery system.

**Supplementary Information:**

The online version contains supplementary material available at 10.1038/s41598-026-53709-8.

## Introduction

Turmeric (*Curcuma longa*) has long been used in traditional medicine. Its major bioactive constituent, curcumin, exerts diverse pharmacological effects, including antioxidant^1^, anti-inflammatory^2^, and neuroprotective activities^3,4^. Recent clinical evidence (2024–2025) confirms curcumin’s therapeutic potential, including cognitive improvements in dementia^5^. According to recent global estimates, approximately 55 million people were living with dementia worldwide in 2019, and this number is projected to increase to 78 million by 2030 and 139 million by 2050, highlighting the growing global burden of neurodegenerative diseases^5^. Despite these promises, curcumin’s clinical utility remains severely limited by poor oral bioavailability^6,7^. Even at 4–8 g/day doses, plasma concentrations rarely exceed 0.41–1.75 µM^8^, which is insufficient for sustained pharmacodynamic activity due to low aqueous solubility, rapid first-pass metabolism, and poor intestinal permeability. This therapeutic gap is particularly relevant in chronic inflammatory and neurodegenerative conditions, where effective systemic exposure is required for sustained pharmacological activity. Various formulation strategies—including phospholipid complexes^6,7^, solid lipid nanoparticles^6,7^, and nanoparticle dispersions^6,7^—have improved bioavailability 5–15-fold. However, conventional single-layer liposomes remain vulnerable to gastric acid degradation, bile-salt solubilization, and enzymatic hydrolysis in the GI tract. In addition, recent work on pH-sensitive PEGylated liposomal prodrugs and gemini surfactant-based nanocarriers has demonstrated that rationally engineered lipid and surfactant systems can substantially enhance systemic exposure and therapeutic performance of small-molecule drugs^6,7,9,10^.

BNT–C060 addresses these limitations through a novel double-layered architecture: a stable phosphatidylcholine nano-liposomal core protected by pH-responsive chitosan/alginate coating. This design simultaneously overcomes curcumin’s three major absorption barriers—chemical instability, poor solubility, and limited permeability. Our prior studies demonstrated 22.2-fold higher C_max_ and 109.5-fold higher AUC_0–t_ in rats^6,7^, plus anti-inflammatory efficacy in PM10-induced lung injury^6,7^. Therefore, the primary aim of this first-in-human study was to test the hypothesis that BNT–C060 achieves substantially higher systemic exposure than free curcumin in healthy volunteers, evaluating plasma/urinary pharmacokinetics and acute safety under fasting conditions as a proof-of-concept for clinical translation.

## Materials and methods

### Chemicals and reagents

Analytical grade curcumin, β-glucuronidase (derived from *Helix pomatia*), and sodium acetate were obtained from Sigma-Aldrich (St. Louis, MO, USA). Employed as the I.S., Curcumin-d6 was obtained from LGC-Standard (Wesel, Germany). Ammonium formate (96.0% purity) was obtained from Samchun Pure Chemical Co., Ltd. (Pyeongtaek, Republic of Korea). HPLC-grade acetonitrile and methanol were obtained from Duksan Pure Chemical Co., Ltd. (Ansan, Republic of Korea). Turmeric extract 95% was obtained from Vidya Herbs Pvt. Ltd (Bangalore, India). Maltodextrin was obtained from Daesang Co., Ltd. (Seoul, Republic of Korea), and ethanol was supplied by Woori Alcohol (Busan, Republic of Korea).

### Ethics

This clinical trial was approved by the Institutional Review Board (IRB) of Wonkwang University Oriental Medical Hospital, Jeonju, Republic of Korea (IRB No. WUJKMH-IRB-2023-0004), and written informed consent was obtained from all participants prior to enrollment. The study was conducted in accordance with the ethical principles of the Declaration of Helsinki and the Korean Good Clinical Practice (KGCP) guidelines. The trial was registered with the Clinical Research Information Service (CRIS), Republic of Korea (Registration No. KCT0009850; protocol identifier: BINO-PK-HC). The study was conducted at Wonkwang University Oriental Medical Hospital (Jeonju, Republic of Korea) and was carried out over a five-month period starting on July 10, 2023.

### Participants

**Fig. 1:**
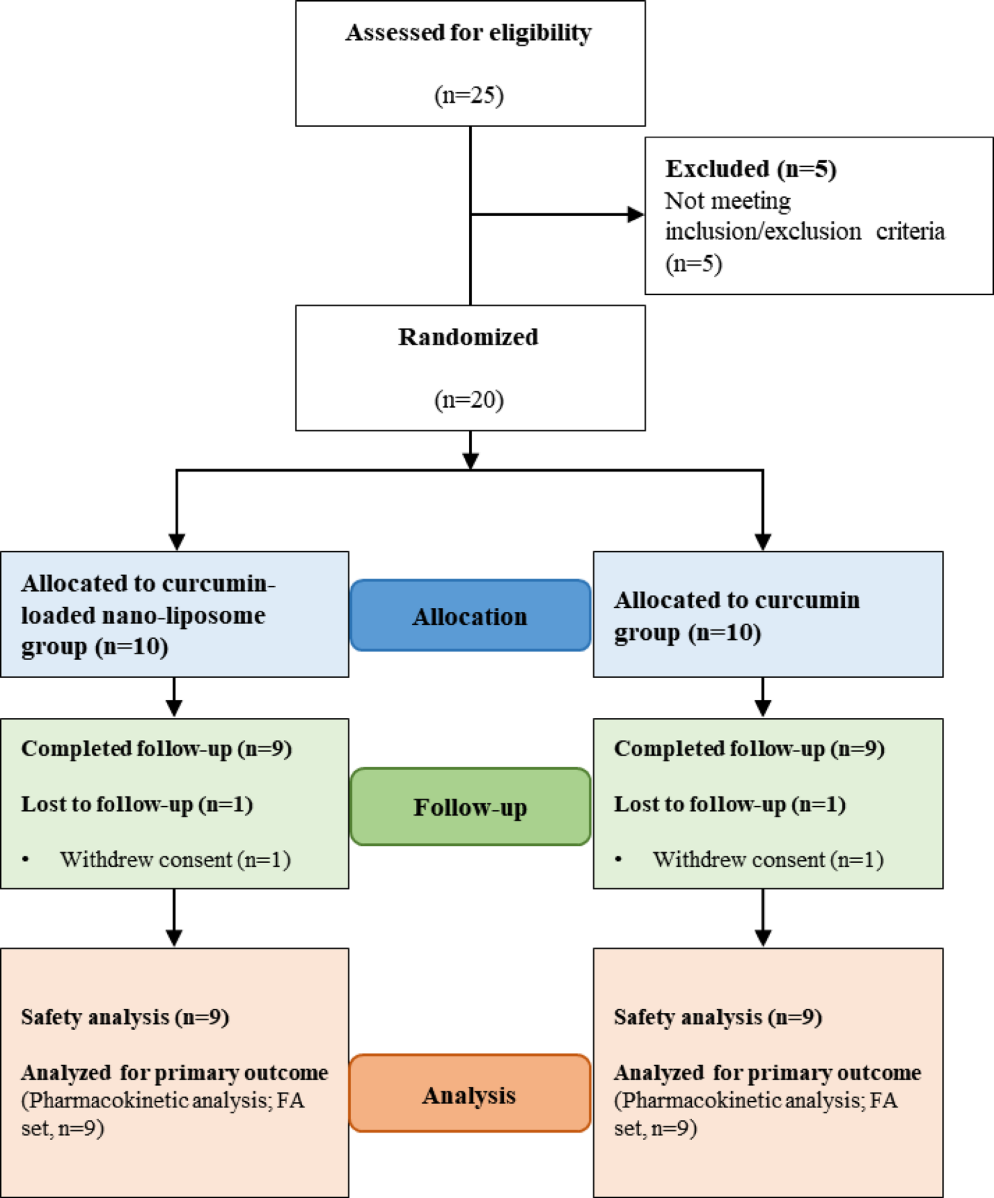
The screening process of the study participants. A total of 25 subjects were recruited for the trial. Of 25 subjects, 18 completed the study (9 in the curcumin-loaded nano-liposome group and 9 in the curcumin group).

Twenty healthy male volunteers participated in this clinical trial, and the participant flow is shown in Fig. 1. To minimize inter-subject variability and avoid the confounding effects of hormonal cycles on gastrointestinal transit and metabolism, only healthy male volunteers were enrolled in this exploratory study. Eligible participants were healthy adults aged 19 to 39 years with a body mass index (BMI) of 17.5–29.9 kg/m² at screening. Exclusion criteria included a history of chronic disease, smoking, use of medication, or intake of curcumin-containing dietary supplements. Female participants were not included in this study because hormonal fluctuations across the menstrual cycle and the possibility of early unrecognized pregnancy may introduce pharmacokinetic variability or safety concerns in early reports^11,12^. The screening procedures included a physical examination, vital signs assessment, blood profiling, medical history review, and biochemical analysis. This clinical study also refers to the trial methodology described by Morimoto et al.^13^. Participants were randomized in a 1:1 ratio to receive either BNT–C060 or free curcumin, using a computer-generated allocation schedule prepared by an independent researcher to ensure balanced baseline characteristics. All doses were administered under fasting conditions to assess the intrinsic pharmacokinetic enhancement of the liposomal formulation, independent of food-induced absorption variability. Baseline demographic and clinical characteristics, including age, body weight, BMI, vital signs, and alcohol consumption, were well-balanced between the treatment groups. Baseline demographic and clinical characteristics of the study participants are summarized in Table 1. Although a statistically significant difference in mean height was observed (176.11 vs. 171.78 cm, *p* = 0.05), the magnitude of this difference was not considered clinically meaningful and was not expected to impact the pharmacokinetic outcome of the study.

**Table 1:** Demographic characteristics of the participants.

Age (years)		BNT–C060 (*n* = 9)	free curcumin (*n* = 9)	Total (*n* = 18)	*p*-value^1)^
		26.11 ± 3.84	28.00 ± 5.33	27.06 ± 4.74	0.428
Height (cm)		176.11 ± 4.68	171.78 ± 3.39	173.94 ± 4.62	**0.050** ^*^
Weight (kg)		75.37 ± 9.09	80.10 ± 9.18	77.73 ± 9.44	0.316
BMI (kg/m ^2^)		22.53 ± 2.53	24.11 ± 2.77	23.32 ± 2.76	0.251
Baseline	SBP (mmHg)	127.67 ± 10.01	125.11 ± 7.17	126.39 ± 8.80	0.565
	DBP (mmHg)	75.11 ± 7.96	74.44 ± 8.86	74.78 ± 8.43	0.876
	Pulse (beat/minute)	67.78 ± 7.19	71.67 ± 8.76	69.72 ± 8.24	0.346
Alcohol (units/week)		5.93 ± 6.74	3.40 ± 3.70	5.15 ± 6.08	0.109
Screening (1 day)	SBP (mmHg)	123.22 ± 7.08	127.22 ± 4.98	125.22 ± 6.44	0.210
	DBP (mmHg)	73.11 ± 7.28	75.11 ± 7.19	74.11 ± 7.30	0.588
	Pulse (beat/minute)	68.89 ± 7.78	70.00 ± 10.18	69.44 ± 9.08	0.809

Values are presented as mean ± SD or number (%). SBP, DBP, and pulse were measured at screening and baseline.^1)^Analyzed by independent t-test between the groups ^*^p < 0.05. signifcant values are given in bold.

### Objectives

The primary objective of this study was to evaluate the systemic bioavailability of curcumin following a single oral dose of BNT–C060 compared with free curcumin in healthy adults, as assessed by plasma pharmacokinetic parameters. Secondary objectives were to characterize urinary pharmacokinetics of curcumin and to assess the safety and tolerability of BNT–C060 under the study conditions.

### Study design, sample size, and underlying rationale

This clinical trial was conducted as a randomized, double-blind, single-dose, parallel-group study to evaluate the pharmacokinetics and bioavailability of curcumin following oral administration of BNT–C060 compared with free curcumin in healthy adults. As no prior human pharmacokinetic data were available for this specific nano-liposomal formulation, a formal confirmatory sample size calculation was not performed. This study was designed as an exploratory, pilot clinical trial. Specifically, this single-dose, parallel-group design was selected as an initial proof-of-concept evaluation to compare the relative bioavailability of BNT–C060 versus free curcumin and establish acute safety/tolerability prior to multiple-dose investigations. Sample size was determined pragmatically to estimate key pharmacokinetic parameters, assess inter-individual variability, and evaluate study feasibility. Early-phase relative bioavailability studies commonly enroll a limited number of participants when the objective is comparative pharmacokinetic characterization rather than formal bioequivalence testing. A total of 20 participants (10 per group) were enrolled, considering an anticipated dropout rate. Eighteen participants (9 per group) completed the study and were included in the pharmacokinetic analyses. This sample size was considered adequate for exploratory estimation of exposure differences and variability to inform the design of future confirmatory studies.

### Randomization and blinding

After screening, participants were randomized 1:1 to receive either BNT–C060 or free curcumin using a computer-generated randomization list prepared by an independent researcher. The random allocation sequence was implemented using sequentially numbered, identical study tubes prepared according to the randomization list, with allocation concealment maintained until the time of dosing.

### Intervention

A new optimized synthetic nano-liposomal formulation of curcumin was prepared as described by Jang et al.^14^. On each dosing day, participants received a single oral dose of either 8 g of BNT–C060 (equivalent to 400 mg of curcumin extract) or 8 g of free curcumin (equivalent to 2,000 mg of curcumin extract) under fasting conditions with 300 mL of mineral water. The curcumin-equivalent doses were determined based on prior consumption experience and available evidence. The difference in administered doses was intentional and designed to explore whether the nano-liposomal formulation could achieve comparable or greater systemic exposure at a lower curcumin-equivalent dose, with comparisons performed using dose-normalized pharmacokinetic parameters. The 400 mg curcumin-equivalent dose of BNT–C060 was selected based on preclinical bridging data and formulation development studies, which indicated that this dose would provide measurable plasma concentrations while maintaining an adequate safety margin for a first-in-human single-dose trial. In contrast, the 2,000 mg dose of free curcumin was chosen to represent a high oral dose commonly employed in previous clinical trials and in marketed products, yet still within a well-established safety range. At such a high dose, free curcumin is likely to be constrained by solubility and intestinal absorptive capacity, potentially resulting in near-saturable uptake. Accordingly, the dose-normalized differences in AUC and C_max_ observed in the present comparison may underestimate the relative bioavailability advantage of BNT–C060 at lower, sub-saturating doses.

### Sample collection and procedures

Blood samples were collected pre-dose (0 min) and at 15, 30, 45, 60, 120, 240, 480, and 1,440 min post-dose. Vital signs—including systolic/diastolic blood pressure, pulse rate, and body temperature were measured at screening (baseline), immediately pre-dose (0 h), and 24 h post-dose (1,440 min) to monitor potential hemodynamic effects. For the pharmacokinetic analysis of curcumin, approximately 5 mL of whole blood was collected from each participant at designated time points into sodium heparinized tubes. To ensure stable plasma separation, the collected blood was kept at room temperature (or on ice, if applicable) and then centrifuged at 3,000 rpm (~ 2,000 × g) for 10 min. The resulting plasma supernatant was immediately transferred to polypropylene tubes and stored at −80 °C until LC–MS/MS analysis. Urine samples were collected over the specified intervals according to the study protocol. Following collection, 5 mL aliquots were transferred into sterile polypropylene tubes. All urine specimens were stored under refrigeration during the collection period and subsequently transferred to a −80 °C freezer for long-term storage prior to analysis. Urine samples were collected pre-dose and over the following post-dose intervals: 0–59, 60–239, 240–479, and 480–1,440 min. Following the completion and verification of the bioanalytical results, all remaining blood and urine samples were disposed of in accordance with the institutional biosafety guidelines and waste management protocols.

### Outcome measures

The primary outcome of this study was systemic exposure to curcumin following a single oral administration of BNT–C060 compared with free curcumin, assessed using plasma pharmacokinetic parameters. The prespecified primary pharmacokinetic endpoints were the maximum plasma concentration (C_max_) and the area under the plasma concentration–time curve from time zero to the last measurable concentration (AUC_0–t_ of total curcumin. Secondary outcomes included additional plasma pharmacokinetic parameters, such as time to maximum concentration (T_max_) and terminal elimination half-life (t_1/2_), as well as urinary pharmacokinetic parameters of total curcumin. Relative bioavailability (F_rel_) of BNT–C060 compared with free curcumin was also evaluated as a secondary outcome. Safety outcomes included the incidence of adverse events, changes in laboratory parameters, and vital signs during the study period.

### Safety assessment

Safety was evaluated as a secondary endpoint from the screening phase until the completion of the 24 h pharmacokinetic assessment. Adverse events (AEs) were monitored systematically through participant interviews, physical examinations, and serial vital sign measurements (blood pressure, pulse rate, and body temperature). To objectively assess the tolerability of both formulations, comprehensive clinical laboratory tests were performed at screening (baseline) and at 24 h post-dose. These evaluations included: (1) Hematology: red blood cell count, white blood cell count, and hemoglobin; (2) Blood chemistry: aspartate aminotransferase (AST), alanine aminotransferase (ALT), and creatinine to monitor hepatic and renal functions; and (3) Urinalysis. All AEs, regardless of causality, were recorded and assessed for clinical significance by the principal investigator. Serious adverse events (SAEs) were predefined according to regulatory criteria and required immediate reporting to the principal investigator and the IRB.

### Analytical condition of LC–MS/MS

Calibration standard samples were prepared by spiking 100 ng/mL curcumin-d6 (I.S.) and different concentrations of curcumin into the blank plasma and urinary sample. The curcumin-related calibration curve calculated using different concentration ranges of curcumin standard (0.2–2,000 ng/mL). The slope, correlation coefficient (r^2^), and intercept of curcumin standard calibration curve measured by the 1/X weighting factor. The linear equation was y = 0.0117x − 0.0073 for plasma, y = 0.0091x + 0.0011 for urine, respectively. All the correlation coefficients (r^2^) exceeded 0.99, confirming the excellent fit of the calibration curve. The LC–MS/MS analysis was performed using an Agilent 1260 Infinity II HPLC system (Agilent Technologies, Santa Clara, CA, USA) coupled with an AB SCIEX Triple Quad 4500 mass spectrometer equipped with a negative electrospray ionization (ESI) source.

Plasma samples were analyzed using an Agilent Poroshell 120 EC-C18 column (2.7 μm, 3.0 × 50 mm) at 25 °C, with the autosampler maintained at 4 °C. The mobile phase consisted of 10 mM ammonium formate in water (A) and methanol (B), applied in a gradient as follows: 0 min, 25% B; 0–3 min, 25–90% B; 3–7.5 min, 90% B; 7.5–7.51 min, 90–25% B; 7.51–11 min, 25% B. The injection volume was 5 µL, and the flow rate was 0.35 mL/min^15^. The mass spectrometric analysis was conducted using an AB SCIEX Triple Quad 4500 system under negative ESI mode. Compounds were detected using multiple reaction monitoring (MRM). Operational parameters were set as follows: collision gas, 9 psi; dissociation gas, 6 psi; desolvation gas, 55 psi; ion spray voltage, −4,500 V; curtain gas, 30 psi; nitrogen as collision gas; and nebulizer temperature, 400 °C. The analyzed processing program was MultiQuant™ (Version 3.0.31721.0; AB SCIEX, Framingham, MA, USA; https://sciex.com/products/software/multiquant-software). Curcumin and Curcumin-d6 (I.S.) monitored by their negative multiple reaction monitoring (MRM) pairs using m/z 367 > 134 and 373 > 134, respectively.

### Plasma and urinary sample preparation

At each time point, 50 µL of plasma or urine was mixed with 55 µL β-glucuronidase solution and incubated at 37 °C for 45 min. After incubation, 50 µL of I.S. and 245 µL of acetonitrile were added. The mixture was vortexed and centrifuged at 10,000 × g for 10 min. The supernatant was filtered through a 0.22-µm syringe filter and subjected to LC–MS/MS analysis.

**Fig. 2:**
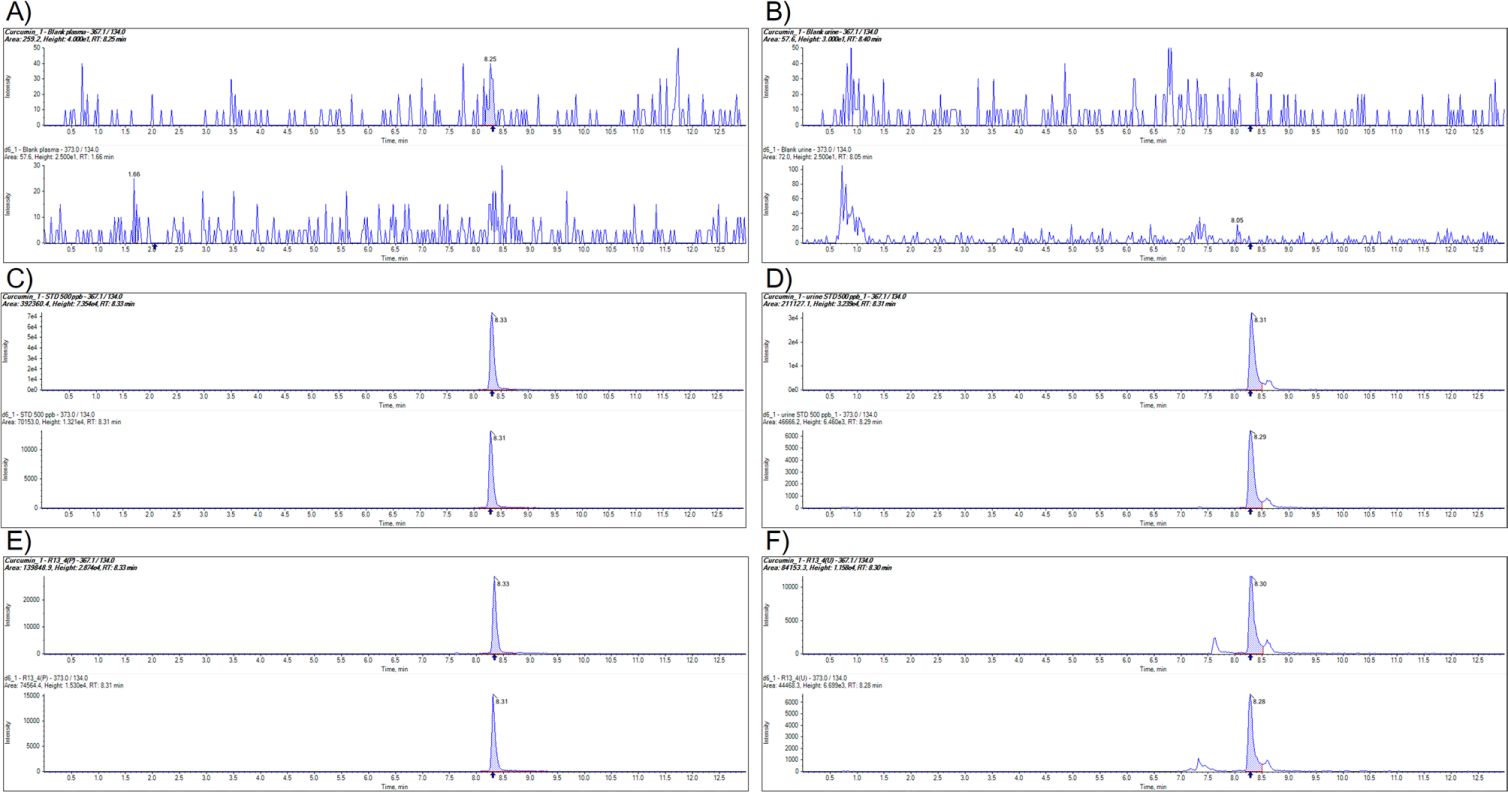
Typical Multiple Reaction Monitoring (MRM) chromatograms of curcumin and curcumin-d6 (I.S.). (**A**) Blank plasma, (**B**) blank urine, (**C**) blank plasma spiked with curcumin and I.S., (**D**) blank urine spiked with curcumin and I.S., (**E**) plasma sample spiked with I.S., (**F**) urine sample spiked with I.S. Representative LC–MS/MS chromatograms of total curcumin (analyte) and curcumin-d6 (internal standard, IS) in plasma samples.

Accordingly, the reported plasma and urinary concentrations represent total curcumin following enzymatic deconjugation. Representative chromatograms are shown in Fig. 2.

### LC–MS/MS analysis of curcumin in plasma and urine

Plasma and urine were prepared using curcumin standard solutions at a range of 0.5–2,000 ng/mL and 0.2–2,000 ng/mL of curcumin concentration, respectively. For the preparation of plasma and urine, blank (50 µL) mixed with curcumin standard solutions (50 µL) at different concentrations, 50 µL of I.S. (100 ng/mL curcumin-d6), 55 µL of 0.1 M sodium acetate buffer (pH 5.0), and 195 µL of acetonitrile. The vortexed mixture was centrifuged at 10,000 × g for 10 min, and the resulting supernatant was filtered through a 0.22 μm PTFE syringe filter prior to analysis.

### Pharmacokinetic and bioavailability of BNT–C060

Plasma and urinary curcumin concentrations were analyzed using a non-compartmental approach. Terminal-phase data from the semi-logarithmic concentration–time curves were subjected to least squares linear regression analysis. Pharmacokinetic parameters were calculated using BA Calc 2007 (Version 1.0.0; Ministry of Food and Drug Safety [MFDS], Seoul, Republic of Korea; https://www.mfds.go.kr) on a personal computer. Maximum plasma and urinary C_max_ and the corresponding T_max_ were determined directly from the observed data. The AUC_0–t_ was calculated from time zero to the last measurable concentration using the linear trapezoidal method. Relative bioavailability (F_rel_ [%]) was calculated as F_rel_= (C_max_ A/Dose A)/(C_max_ B/Dose B) or F_rel_= (AUC_0–t_ A/Dose A)/(AUC_0–t_ B/Dose B), where A is BNT–C060 and B is free curcumin.

### Statistical analysis

This pilot study performed no formal confirmatory hypothesis testing. Due to the small sample size (*n* = 9/group) and exploratory design, all p-values are reported solely for descriptive and hypothesis-generating purposes to estimate effect magnitudes and inform future confirmatory trial design. No analyses were intended to support definitive statistical claims or regulatory decisions. No formal power calculation or multiplicity adjustments were performed; results should be interpreted as trends rather than formal statistical conclusions. Statistical analyses were performed using BA Calc 2007 (NCA) or SAS for Windows. All tests were two-sided, with *p* < 0.05 considered statistically significant for descriptive purposes only. Pharmacokinetic analyses were performed in the full analysis set (FA set), defined as all randomized participants who received the study product and had at least one quantifiable post-dose plasma concentration of curcumin without major protocol deviations affecting pharmacokinetic evaluation, and safety analyses were conducted in the Safety population (participants who received at least one dose).

Baseline characteristics were compared using the Chi-square or Fisher’s exact test for categorical variables and the independent t-test for continuous variables as descriptive balancing checks. Pharmacokinetic parameters (C_max_, T_max_, AUC_0–t_, and relative bioavailability [F_rel_]) were summarized descriptively by treatment group. Post-hoc normality assessment (Shapiro-Wilk test, *p* > 0.05) confirmed that dose-normalized AUC_0–t_ and C_max_ exhibited acceptable normality in both groups. Therefore, dose-normalized AUC_0–t_ and C_max_ were compared between groups using independent t-tests as exploratory analyses (not confirmatory testing) to quantify bioavailability differences, per standard practice for relative bioavailability studies (FDA 2003). T_max_ was compared between groups using Wilcoxon rank-sum tests as prespecified in the protocol (Sect. 5.10.3), with results reported as medians and interquartile ranges per standard PK convention for descriptive purposes. Other secondary parameters (t_1/2_, F_rel_) were summarized descriptively, and p-values were provided for descriptive purposes only in this exploratory study. AEs were summarized as frequencies/percentages and compared using the Chi-square or Fisher’s exact test descriptively. Laboratory tests and vital signs were summarized descriptively; within-group changes from baseline to 24 h post-dose were descriptively assessed using paired t-tests for hematology parameters (red blood cell count, white blood cell count, hemoglobin), blood chemistry (AST, ALT, creatinine), and vital signs (systolic/diastolic blood pressure, pulse rate, body temperature); and between-group differences in change-from-baseline were descriptively assessed using the independent t-test.

Accordingly, all p-values are presented for descriptive purposes and should not be interpreted as evidence of inferential statistical significance.

## Results

### Participants

Participant recruitment was conducted between July 2023 and December 2023. A total of 25 individuals were assessed for eligibility, of whom 20 were randomized and assigned in a 1:1 ratio to receive either BNT–C060 or free curcumin (*n* = 10 per group). Five individuals were excluded prior to randomization due to not meeting the inclusion or exclusion criteria. The flow of participants through the study is shown in Fig. 1. All randomized participants received the assigned intervention as intended. During follow-up, one participant in each group withdrew consent and was lost to follow-up, resulting in 9 participants per group completing the study. Accordingly, safety analyses were conducted in 18 participants (9 per group), and pharmacokinetic analyses were performed in the full analysis set (FA set; *n* = 9 per group), defined as participants with at least one quantifiable post-dose plasma concentration of total curcumin. Follow-up for pharmacokinetic and safety outcomes was completed within 24 h after dosing for all participants included in the analyses. The trial was completed as planned, with no early termination. No concomitant medications or additional medical interventions were administered during the study period, and all participants received identical standardized meals and controlled water intake during hospitalization.

### Safety assessments of BNT–C060

Participants received either 8 g of BNT–C060 (equivalent to 400 mg curcumin extract) or 8 g of free curcumin (equivalent to 2,000 mg curcumin extract). No unexpected adverse events were observed in either group (data not shown). Although minor fluctuations in alkaline phosphatase and hemoglobin levels occurred, all measurements remained within normal clinical ranges and were not clinically significant. Other blood chemistry values, hematological parameters, and vital signs remained unremarkable. These results indicate that BNT–C060 is safe and well tolerated at the administered dose levels, consistent with the report by Belcaro et al., who found no significant adverse effects after administering 1,000 mg of a curcumin formulation (equivalent to 200 mg curcumin) in humans^16^. Safety assessments revealed no clinically meaningful changes in vital signs from pre-dose to 24 h post-dose; changes in systolic blood pressure (−2.1 ± 8.3 mmHg for BNT–C060 vs. 1.2 ± 7.9 mmHg for free curcumin) and diastolic blood pressure (0.3 ± 5.1 vs. −1.1 ± 6.2 mmHg, respectively) were small and not statistically notable (all *p* > 0.30).

### Pharmacokinetic analysis of BNT–C060 in plasma

**Fig. 3:**
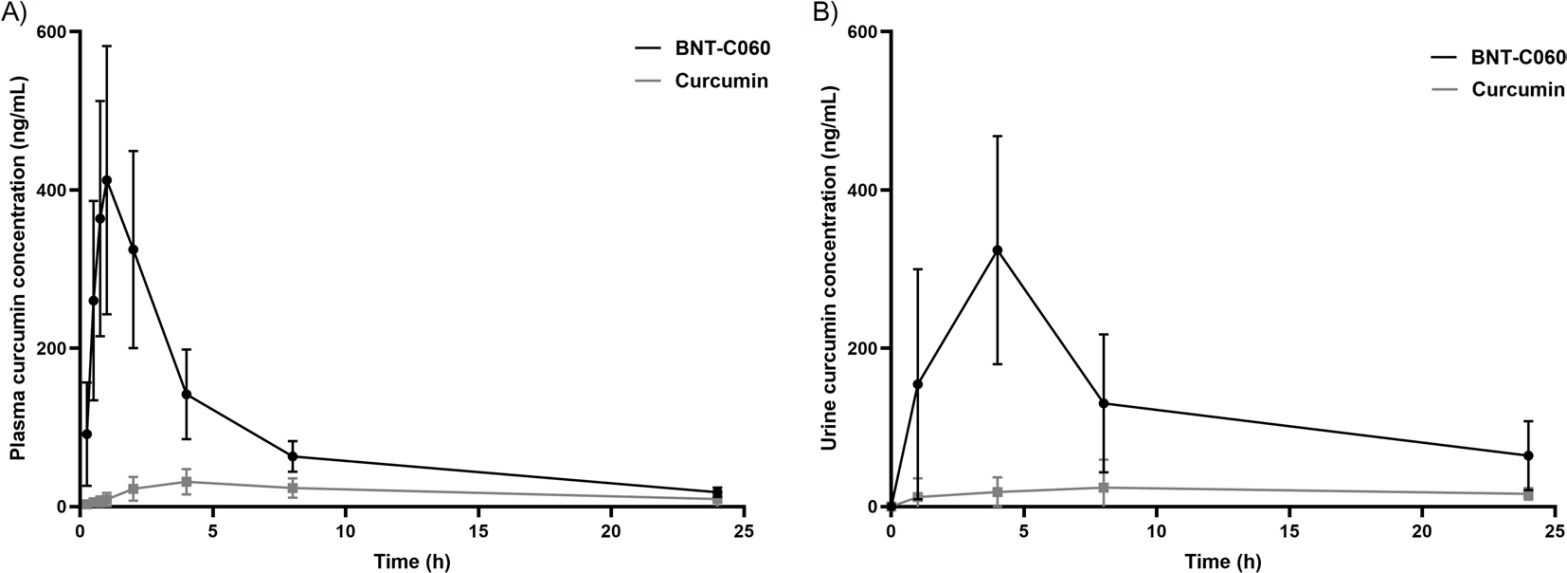
The mean total curcumin concentration-time profiles in human plasma and urine. (**A**) Plasma curcumin concentration-time profile, (**B**) urine curcumin concentration-time profile.

**Fig. 4:**
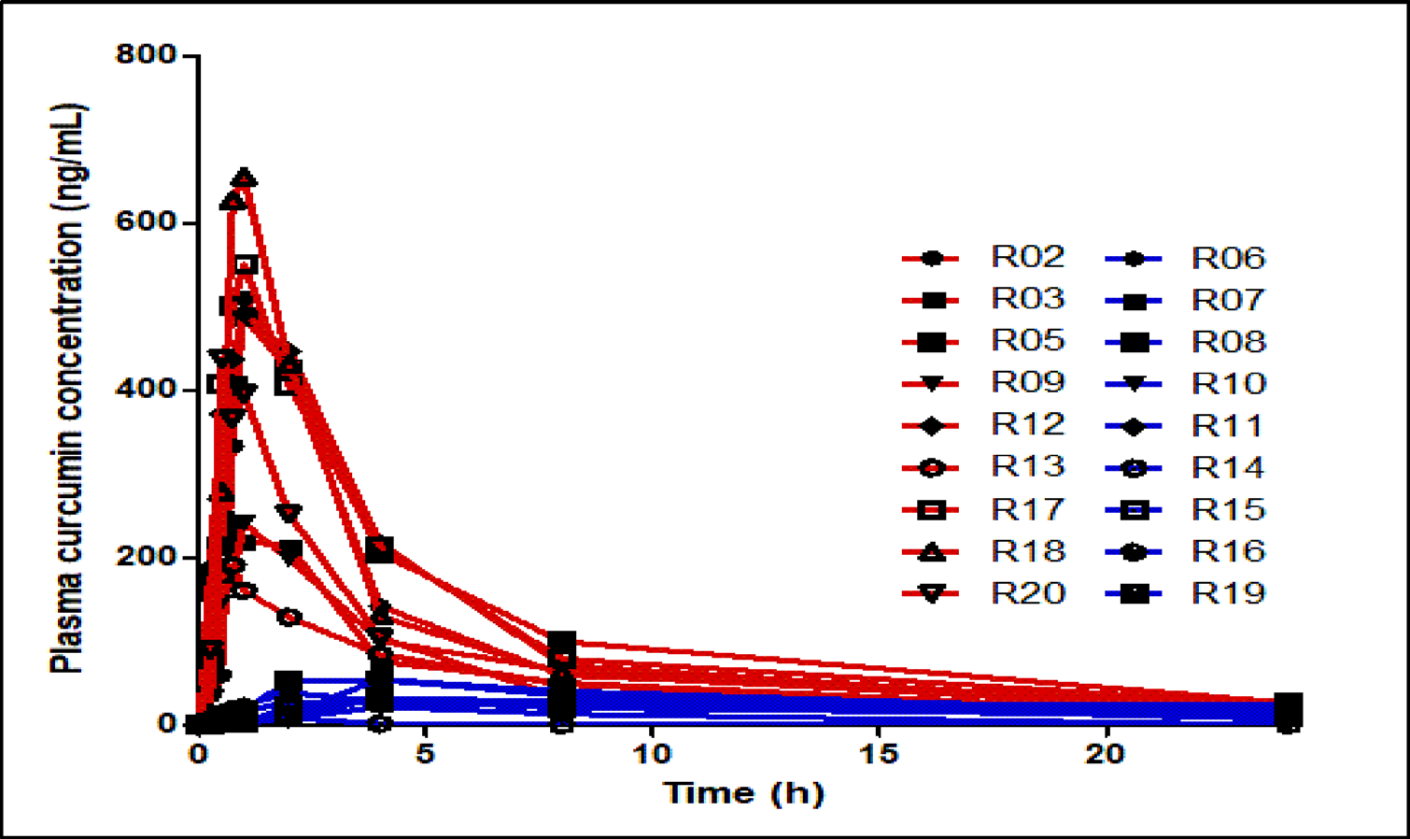
Individual plasma and urinary concentration–time profiles of curcumin following single-dose administration of BNT–C060 and free curcumin. (**A**) Plasma curcumin concentrations (ng/mL) and (**B**) urinary curcumin concentrations (ng/mL) are shown for each individual participant over 24 h following oral administration of BNT–C060 (400 mg curcumin-equivalent; participants R02–R20: Red line) or free curcumin extract (2,000 mg; participants R06–R19: Blue line) under fasting conditions. Each line represents an individual participant. The plots illustrate the concentration–time profiles and inter-individual variability in systemic exposure between the two formulations. Note: Importantly, most participants receiving BNT–C060 exhibited substantially higher plasma concentrations throughout the sampling period compared with the free curcumin group.

Pharmacokinetic comparisons between BNT–C060 (curcumin-equivalent dose: 400 mg) and free curcumin (2,000 mg) were interpreted using dose-normalized parameters to account for the 5-fold difference in administered curcumin content. When dose-normalized, BNT–C060 demonstrated markedly higher AUC_0–t_ (5.00 ± 1.54 vs. 0.22 ± 0.11 ng·h/mL/mg) and C_max_ (1.06 ± 0.40 vs. 0.02 ± 0.01 ng/mL/mg) than free curcumin (Table 2). Pharmacokinetic parameters were measured following a single oral administration of BNT–C060 or free curcumin, with plasma concentrations of total curcumin quantified using LC–MS/MS (Table 2; Fig. 3A). The plasma AUC_0–t_ of the BNT–C060 group was 2,001.50 ± 616.35 ng·h/mL, demonstrating a notable increase compared with free curcumin (431.34 ± 227.89 ng·h/mL, *p* < 0.05). The C_max_ also showed a marked elevation in the BNT–C060 group (423.41 ± 160.03 ng/mL) compared with free curcumin (33.50 ± 14.64 ng/mL, *p* < 0.05). T_max_ was earlier in the BNT–C060 group (median 0.86 h, IQR 0.50–1.0.50.0) than in the free curcumin group (median 3.3 h, IQR 2.0–4.0; Wilcoxon rank-sum test, *p* = 0.008), consistent with more rapid absorption of the nano-liposomal formulation. The elimination half-life (t_1/2_) was 6.74 h for BNT–C060 and 24.92 h for free curcumin; however, the apparently prolonged t_1/2_ in the free curcumin group should be interpreted cautiously because late-phase concentrations approached the lower quantifiable range. To further evaluate inter-individual variability, individual plasma and urinary concentration–time profiles are presented in Fig. 4, and detailed participant-level pharmacokinetic parameters, including dose-normalized values, are provided in Supplemental Table S1. The individual concentration–time profiles demonstrate that systemic exposure was consistently higher in the BNT–C060 group compared with free curcumin, despite observable inter-subject variability typical of early-phase pharmacokinetic studies. When expressed as effect sizes, the geometric mean ratio (BNT–C060/free curcumin) for dose-normalized AUC_0–t_ was 23.2, and for dose-normalized C_max_ was 53.0, confirming that BNT–C060 produced a several-fold enhancement in systemic exposure compared with free curcumin.

**Table 2:** Pharmacokinetic parameters of total curcumin in plasma and urine following oral administration of BNT–C060 and free curcumin.

Parameters	Plasma		Urine	
	BNT–C060	Free curcumin	BNT–C060	free curcumin
Curcumin-equivalent dose (mg)	400	2000	400	2000
AUC_0–t_ (ng·h/mL)	2001.50 ± 616.35	431.34 ± 227.89	3067.96 ± 1,291.52	434.76 ± 404.82
Dose-normalized AUC_0–t_ (ng·h/mL/mg)	5.00 ± 1.54	0.22 ± 0.11	7.67 ± 3.23	0.22 ± 0.20
C_max_ (ng/mL)	423.41 ± 160.03	33.50 ± 14.64	324.13 ± 144.01	36.17 ± 36.29
Dose-normalized C_max_ (ng/mL/mg)	1.06 ± 0.40	0.02 ± 0.01	0.81 ± 0.36	0.02 ± 0.02
T_max_ (h)	0.86 (0.50–1.00.50.00)	3.30 (2.00–4.00)	4.00 (2.00–6.00)	13.00 (8.00–24.00)
t_1/2_ (h)	6.74	24.92	22.28	21.70
F_rel_ (fold)	23.20	-	35.28	-

### Pharmacokinetic analysis of BNT–C060 in urine

Urinary pharmacokinetic profiles were also assessed (Table 2; Fig. 3B). The AUC_0–t_ of BNT–C060 (3,067.96 ± 1,291.52 ng·h/mL) was substantially greater than that of free curcumin (434.76 ± 404.82 ng·h/mL, *p* < 0.001). The C_max_ was higher in the BNT–C060 group (324.13 ± 144.01 ng/mL) than in the free curcumin group (36.17 ± 36.29 ng/mL, *p* < 0.05). The T_max_ was earlier in the BNT–C060 group (4 h) compared with free curcumin (13 h). No significant differences were observed in the elimination half-life between groups.

### Relative bioavailability

The relative bioavailability (F_rel_) of BNT–C060 was markedly enhanced compared with free curcumin. In plasma, F_rel_ increased 23.2-fold, and in urine, 35.28-fold (Table 2). These data suggest a substantial increase in systemic exposure following oral administration of the double-layered nano-liposomal formulation.

## Discussion

This clinical study indicates that BNT–C060, a double-layered chitosan/alginate-coated nano-liposomal curcumin formulation, was associated with higher oral exposure to curcumin than free curcumin, without clinically meaningful short-term safety concerns under the conditions tested. Importantly, inspection of individual participant-level data (Fig. 4; Supplemental Table S1) indicates that the enhanced exposure observed with BNT–C060 was not driven by a single outlier but reflected a group-level trend. No clinically relevant adverse effects were identified, consistent with prior reports showing the safety of curcumin formulations at comparable doses^16^. Importantly, these data reflect single-dose pharmacokinetics only. Multiple-dose studies will be required to assess steady-state exposure, potential drug accumulation, and time-dependent changes in bioavailability that may occur with repeated administration.

### Pharmacokinetic comparison with previously reported formulations

A key finding is the markedly higher plasma AUC_0–t_ and C_max_ achieved by BNT–C060. In earlier studies, curcumin-loaded delivery systems such as solid lipid curcumin phospholipid complexes (SLCPs) produced AUC values of approximately 95.3 ng·h/mL and C_max_ values around 22.4 ng/mL^17^. Gum ghatti-based nanoparticle dispersions yielded AUC_0−t_ values of 113 ng·h/mL and C_max_ values around 29.5 ng/mL^18^, while phospholipid-based phytosomal formulations achieved higher exposure (AUC_0–t_ 538 ng·h/mL and C_max_ 50.3 ng/mL)^19^. In contrast, BNT–C060 produced an AUC_0–t_ of 2,001.50 ng·h/mL and C_max_ of 423.41 ng/mL, which appears higher than the numerical values reported for several advanced curcumin delivery technologies. Notably, these delivery platforms serve as the basis for several well-known marketed supplements, such as Meriva^®^ (phytosome), Longvida^®^ (solid lipid particles), and Theracurmin^®^ (colloidal dispersion). Although direct cross-study comparisons are limited by differences in dose, formulation, and analytical methodology, the exposure levels observed with BNT–C060 are numerically comparable to those reported for several commercially available curcumin delivery systems.

### Mechanistic basis of the double-layered architecture

**Fig. 5:**
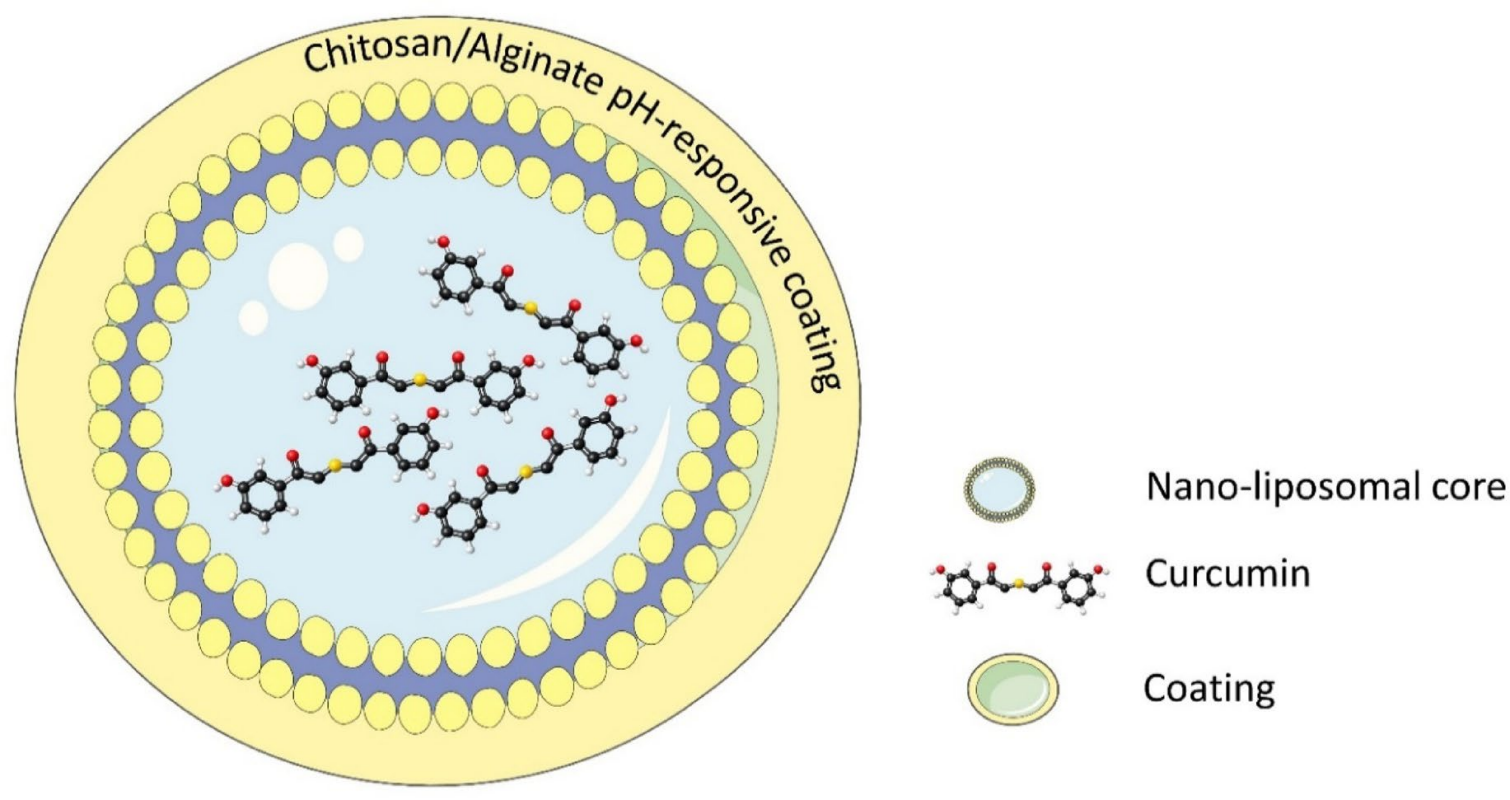
Schematic illustration of the double-layered nano-liposomal structure of BNT–C060. The formulation consists of a phosphatidylcholine-based nano-liposomal core encapsulating curcumin and an outer chitosan/alginate polymer coating. This double-layered architecture provides pH-responsive protection and mucoadhesive properties that help maintain liposomal stability and support intestinal absorption of curcumin.

The mechanistic basis for the improved bioavailability of BNT–C060 is illustrated in Fig. 5. The enhanced pharmacokinetic profile reflects the double-layered architecture of BNT–C060, which may help address three major limitations of conventional single-layer liposomes. Firstly, gastric degradation: Single-layer liposomes disintegrate rapidly at pH 1.5–3.5 (half-life < 30 min), precipitating curcumin. Chitosan/alginate coating remains stable below pH 4.5, dissolving selectively at intestinal pH (> 6.5)^14^. Secondly, bile salt solubilization: Duodenal bile salts destroy phospholipid bilayers. Cationic chitosan provides electrostatic repulsion against anionic bile salts, maintaining structural integrity. Thirdly, rapid transit: Standard liposomes exhibit poor mucoadhesion (< 2 h residence). Chitosan enhances mucoadhesion, prolongs gastrointestinal residence^20^, and may facilitate paracellular absorption through transient tight junction modulation^21^. In addition, chitosan coating improves the gastric stability of curcumin-loaded liposomes and helps retain curcumin under simulated gastric conditions compared with uncoated formulations^14^, which may partly contribute to the observed 23.2-fold increase in dose-normalized bioavailability. This triple-protection strategy, combined with a dose-sparing effect (400 mg vs. 2,000 mg equivalents), supports the potential pharmacokinetic advantages of BNT–C060 relative to previously reported curcumin formulations. Collectively, these properties may help explain the improved pharmacokinetic performance of BNT–C060. The shorter T_max_ observed with BNT–C060 is consistent with more rapid and efficient intestinal uptake. By contrast, the apparent prolongation of t_1/2_ in the free curcumin group is more likely to reflect uncertainty in terminal-phase estimation at concentrations near the LLOQ than a true difference in elimination kinetics. In addition, flip-flop kinetics cannot be excluded in the free curcumin group because of its poor solubility and potentially prolonged absorption.

### Translational and pharmacodynamic implications

In the broader context of nanotechnology-enhanced curcumin delivery, previously reported polymeric micelles and nano-emulsions increased bioavailability by approximately 9.7–15.8-fold^22,23^. In the present study, BNT–C060 achieved a 23.2-fold (plasma) and 35.3-fold (urine) increase in dose-normalized relative bioavailability (F_rel_). One possible explanation for the translational relevance of this pharmacokinetic enhancement is a threshold-exposure hypothesis. Curcumin has been reported to exert anti-inflammatory and antioxidant effects through inhibition of NF-κB signaling, suppression of pro-inflammatory cytokines such as TNF-α, IL-6, and IL-1β, and activation of Nrf2-associated pathways^1^. Accordingly, the substantially higher systemic exposure achieved with BNT–C060 may increase the likelihood of reaching biologically relevant concentrations compared with free curcumin. These marked improvements in systemic exposure may have pharmacodynamic relevance; however, this exploratory pharmacokinetic study was not designed to directly evaluate pharmacodynamic effects. Therefore, the relationship between the observed pharmacokinetic enhancement and clinical efficacy remains to be established in future trials.

In this study, urinary total curcumin was evaluated as a supportive marker of systemic exposure rather than as a primary elimination pathway, because curcumin is predominantly excreted via biliary and fecal routes. After β-glucuronidase treatment, the measured urinary concentrations reflect total deconjugated curcumin species and therefore provide complementary information on absorbed and subsequently metabolized curcumin, rather than on renal clearance of the unchanged parent compound. Previous preclinical studies have suggested that improved oral delivery of BNT–C060 may enhance the biological activity of curcumin at relatively low doses^24^. In the context of the present study, these findings support the pharmacological relevance of improving systemic exposure, although direct pharmacodynamic effects were not evaluated here.

This pilot PK study was conducted under strictly fasting conditions to reduce inter-individual variability and to isolate the formulation-driven contribution of BNT–C060 to oral bioavailability, independent of food effects. High-fat meals are known to increase curcumin exposure by enhancing micellar solubilization, but it remains uncertain to what extent fed conditions would differentially affect free curcumin and a double-layered nano-liposomal system such as BNT–C060. Under fed conditions, both formulations are likely to show increased exposure; however, BNT–C060 may be less dependent on dietary fat owing to carrier-mediated solubilization, whereas free curcumin may benefit more directly from fat-induced bile secretion. As the present data are limited to fasting conditions, future studies including formal fed–fasting comparisons will be required to fully characterize the bioavailability profile of BNT–C060 in real-world dietary settings.

Because this trial was exploratory in nature, no formal a priori sample size calculation was performed. Nevertheless, the observed effect sizes and variability in dose-normalized exposure provide empirical estimates for planning future confirmatory trials. In this study, dose-normalized AUC_0–t_ was 5.00 ± 1.54 ng·h·mL⁻¹·mg⁻¹ with BNT–C060 and 0.22 ± 0.11 ng·h·mL⁻¹·mg⁻¹ with free curcumin, corresponding to a 23.2-fold increase and coefficients of variation of approximately 31% and 50%, respectively. Dose-normalized C_max_ values were 1.06 ± 0.40 and 0.02 ± 0.01 ng·mL⁻¹·mg⁻¹ for BNT–C060 and free curcumin, indicating a 53-fold difference with CVs of about 38% and 50%. These effect size and variability estimates can serve as input parameters for future power calculations to determine the number of participants required to detect prespecified relative bioavailability differences between BNT–C060 and comparator formulations with adequate statistical power.

Despite these encouraging findings, several limitations should be acknowledged. First, the sample size was small and designed for exploratory pharmacokinetic assessment rather than confirmatory hypothesis testing, which may limit generalizability. Second, only healthy male volunteers were included. Thus, sex-specific or disease-related variability could not be assessed. Third, curcumin concentrations were quantified following β-glucuronidase treatment and therefore represent total curcumin (free plus conjugated forms), precluding precise evaluation of unconjugated pharmacologically active curcumin. In addition, potential differences in first-pass metabolism, enterohepatic recycling, and inter-individual metabolic variability, including possible genetic polymorphisms, may have contributed to the observed pharmacokinetic differences. Beyond these methodological considerations, further studies are required to evaluate long-term safety, multiple-dose pharmacokinetics, food effects, and pharmacodynamic responses in relevant patient populations. In addition, manufacturing scalability, formulation stability, and regulatory classification considerations will be important for future clinical translation.

In conclusion, this randomized, double-blind, single-dose clinical trial showed that BNT–C060 markedly enhances systemic exposure to curcumin compared with free curcumin under fasting conditions in healthy male volunteers. Dose-normalized analysis showed a 23.2-fold higher AUC_0–t_ and a 53-fold higher C_max_, accompanied by earlier T_max_ and increased urinary exposure, without clinically meaningful safety concerns. A single oral dose of BNT–C060 was safe and well tolerated in healthy male volunteers within the evaluated dose range. These findings provide human proof-of-concept that the double-layered chitosan/alginate-coated nano-liposomal architecture improves curcumin delivery efficiency. Although the study was exploratory and limited to a small cohort under single-dose conditions, the observed enhancement in bioavailability supports further investigation in multiple-dose studies and more diverse populations to clarify pharmacodynamic relevance and long-term safety. Collectively, these results establish a translational foundation for subsequent clinical evaluation in broader populations and study settings.

## Supplementary Information

Below is the link to the electronic supplementary material.

Supplementary Material 1

## Data Availability

The study protocols, datasets, statistical analysis plan are available from the corresponding author upon reasonable request.
