# Supplementary material for: Enhanced bioavailability of a novel double-layered nano-liposomal curcumin (BNT–C060): a randomized, double-blind, clinical trial
Source: Sci Rep. 2026 May 31;16:24863. doi: 10.1038/s41598-026-53709-8 (PMC13457880; doi:10.1038/s41598-026-53709-8)
Supplement: Supplementary file 1 [file 41598_2026_53709_MOESM1_ESM.docx]

**SUPPLEMENTARY TABLES**

**Statistical Analysis of Pharmacokinetic Parameters**

**Supplemental Table S1. Individual pharmacokinetic parameters following single-dose administration of BNT–C060 and free curcumin.**

Participant-level pharmacokinetic parameters, including C_max_, T_max_, and AUC_0–24_, are presented for each individual in both treatment groups (n = 9 per group). Dose-normalized values were calculated by dividing parameters by the administered curcumin-equivalent dose (400 mg for BNT–C060; 2,000 mg for free curcumin) to allow direct evaluation of inter-individual variability. Data are expressed as individual values and mean. Abbreviations: AUC_0–24_, area under the concentration–time curve from 0 to 24 h; C_max_, maximum observed concentration; T_max_, time to reach maximum concentration.

**A) Plasma Pharmacokinetic Parameters**

| Subject ID | Group | C_max_ (ng/mL) | T_max_  (h) | AUC_0–24_ (ng·h/mL) | C_max_ /Dose | AUC_0–24_ /Dose |
| --- | --- | --- | --- | --- | --- | --- |
| R02 | BNT–C060 | 510.2 | 1 | 2289.9 | 1.276 | 5.725 |
| R03 | BNT–C060 | 246.7 | 0.5 | 1448.1 | 0.617 | 3.62 |
| R05 | BNT–C060 | 486.7 | 1 | 2968.3 | 1.217 | 7.421 |
| R09 | BNT–C060 | 241 | 1 | 1640.9 | 0.603 | 4.102 |
| R12 | BNT–C060 | 491.6 | 1 | 2729.1 | 1.229 | 6.823 |
| R13 | BNT–C060 | 191.1 | 0.75 | 1225.6 | 0.478 | 3.064 |
| R17 | BNT–C060 | 550.4 | 1 | 2824.6 | 1.376 | 7.061 |
| R18 | BNT–C060 | 655.6 | 1 | 2420.2 | 1.639 | 6.05 |
| R20 | BNT–C060 | 437.6 | 0.5 | 1617.3 | 1.094 | 4.043 |
| Mean | BNT–C060 | 423.4 | 0.86 | 2129.3 | 1.059 | 5.323 |
| R06 | Free Curcumin | 38.9 | 2 | 472 | 0.019 | 0.236 |
| R07 | Free Curcumin | 28.6 | 2 | 572.9 | 0.014 | 0.286 |
| R08 | Free Curcumin | 54.4 | 4 | 687.3 | 0.027 | 0.344 |
| R10 | Free Curcumin | 21.8 | 4 | 217.6 | 0.011 | 0.109 |
| R11 | Free Curcumin | 31.3 | 4 | 473.2 | 0.016 | 0.237 |
| R14 | Free Curcumin | 8.7 | 2 | 22.1 | 0.004 | 0.011 |
| R15 | Free Curcumin | 29.9 | 4 | 430.1 | 0.015 | 0.215 |
| R16 | Free Curcumin | 54.9 | 4 | 711.2 | 0.027 | 0.356 |
| R19 | Free Curcumin | 32.9 | 4 | 562.3 | 0.016 | 0.281 |
| Mean | Free Curcumin | 33.5 | 3.33 | 461 | 0.017 | 0.23 |

**B) Urinary Pharmacokinetic Parameters**

| Subject ID | Group | C_max_ (ng/mL) | T_max_  (h) | AUC_0–24_ (ng·h/mL) | C_max_ /Dose | AUC_0–24_ /Dose |
| --- | --- | --- | --- | --- | --- | --- |
| R02 | BNT–C060 | 353.6 | 4 | 2871.8 | 0.884 | 7.18 |
| R03 | BNT–C060 | 534.2 | 4 | 6127.1 | 1.336 | 15.318 |
| R05 | BNT–C060 | 171.5 | 4 | 2620.7 | 0.429 | 6.552 |
| R09 | BNT–C060 | 171.5 | 4 | 1321.1 | 0.429 | 3.303 |
| R12 | BNT–C060 | 253.3 | 4 | 2334 | 0.633 | 5.837 |
| R13 | BNT–C060 | 239.6 | 4 | 3267.8 | 0.599 | 8.17 |
| R17 | BNT–C060 | 239.4 | 4 | 2333.1 | 0.599 | 5.833 |
| R18 | BNT–C060 | 412.2 | 4 | 3892.7 | 1.031 | 9.732 |
| R20 | BNT–C060 | 541.8 | 4 | 4612.1 | 1.355 | 11.53 |
| Mean | BNT–C060 | 324.1 | 4 | 3264.6 | 0.81 | 8.161 |
| R06 | Free Curcumin | 17 | 4 | 334.7 | 0.009 | 0.167 |
| R07 | Free Curcumin | 24.5 | 4 | 510.6 | 0.012 | 0.255 |
| R08 | Free Curcumin | 36.7 | 4 | 453.1 | 0.018 | 0.227 |
| R10 | Free Curcumin | 75.1 | 1 | 352.6 | 0.038 | 0.176 |
| R11 | Free Curcumin | 8.2 | 24 | 117.9 | 0.004 | 0.059 |
| R14 | Free Curcumin | 12.9 | 24 | 156.9 | 0.006 | 0.078 |
| R15 | Free Curcumin | 24.3 | 24 | 432.2 | 0.012 | 0.216 |
| R16 | Free Curcumin | 10.7 | 24 | 157.1 | 0.005 | 0.079 |
| R19 | Free Curcumin | 116.2 | 8 | 1605 | 0.058 | 0.803 |
| Mean | Free Curcumin | 36.2 | 13 | 457.8 | 0.018 | 0.229 |

**Supplemental Table 2. Statistical comparison of p-values for Plasma AUC_0–t_ and Plasma C_max_ using different analysis methods.** Data show the statistical significance (p-values) from the independent t-test, Log-transformed t-test, and linear model analysis. All values are less than 0.05, indicating significant differences across all tested models.

| **Analysis method** | **Plasma AUC_0–t_ (p-value)** | **Plasma C_max_ (p-value)** |
| --- | --- | --- |
| **Independent t-test** | 0.002 | 0.001 |
| **Log-transformed t-test** | 0.003 | 0.002 |
| **Linear model** | 0.002 | 0.001 |

**Supplemental Table S3. Comparative analysis of T_max_ between BNT–C060 and Free Curcumin (n=9 per group).** The table presents the median, interquartile range (IQR), range, and individual values for time to maximum concentration (T_max_). Statistical comparison was performed using the Wilcoxon rank-sum test. Individual participant values are listed to illustrate the distribution of absorption timing.

| **Parameter** | **BNT–C060** | **Free Curcumin** | **Wilcoxon rank-sum test** |
| --- | --- | --- | --- |
| **Median T_max_ (h)** | **1** | **4** | **p < 0.001** |
| IQR (h) | 0.75–1.0 | 2.0–4.0 |  |
| Min-Max (h) | 0.5–1.0 | 2.0–4.0 |  |
| Individual values (h) | 0.5, 0.5, 0.75, 1.0, 1.0, 1.0, 1.0, 1.0, 1.0 | 2.0, 2.0, 2.0, 4.0, 4.0, 4.0, 4.0, 4.0, 4.0 |  |

**Notes:**
- T_max_ comparisons performed using Wilcoxon rank-sum test as prespecified in protocol Section 5.10.3.
- p < 0.001 indicates statistically significant shorter T_max_ for BNT–C060.
- All BNT–C060 T_max_ values ≤1.0 h; all free curcumin T_max_ values ≥1.0 h (no overlap).
